# Metabolomic insights into maternal and neonatal complications in pregnancies affected by type 1 diabetes

**DOI:** 10.1007/s00125-023-05989-2

**Published:** 2023-08-24

**Authors:** Claire L. Meek, Zoe A. Stewart, Denice S. Feig, Samuel Furse, Sandra L. Neoh, Albert Koulman, Helen R. Murphy, on behalf of the CONCEPTT collaborative group

**Affiliations:** 1 Wellcome-MRC Institute of Metabolic Science, University of Cambridge, Cambridge, UK; 2 Cambridge University Hospitals NHS Foundation Trust, Cambridge, UK; 3 Department of Cardiovascular Sciences, University of Leicester, Leicester, UK; 4 Mount Sinai Hospital, Sinai Health System, New York, NY USA; 5 Department of Medicine, University of Toronto, Toronto, ON Canada; 6 Lunenfeld-Tanenbaum Research Institute, Toronto, ON Canada; 7 Core Metabolomics and Lipidomics Laboratory, Institute of Metabolic Science, University of Cambridge, Cambridge, UK; 8 Department of Endocrinology, Austin Health, Melbourne, VIC Australia; 9 Department of Endocrinology, Northern Health, Melbourne, VIC Australia; 10 Norwich Medical School, University of East Anglia, Norwich, UK

**Keywords:** Cord blood, LGA, Lipidomics, Metabolomics, Neonatal hypoglycaemia, Prediction, Pre-eclampsia, Pregnancy, Pregnancy complications, Pregnancy in people with diabetes, Pregnancy outcome, Type 1 diabetes

## Abstract

**Aims/hypothesis:**

Type 1 diabetes in pregnancy is associated with suboptimal pregnancy outcomes, attributed to maternal hyperglycaemia and offspring hyperinsulinism (quantifiable by cord blood C-peptide). We assessed metabolomic patterns associated with risk factors (maternal hyperglycaemia, diet, BMI, weight gain) and perinatal complications (pre-eclampsia, large for gestational age [LGA], neonatal hypoglycaemia, hyperinsulinism) in the Continuous Glucose Monitoring in Women with Type 1 Diabetes in Pregnancy Trial (CONCEPTT).

**Methods:**

A total of 174 CONCEPTT participants gave ≥1 non-fasting serum sample for the biorepository at 12 gestational weeks (147 women), 24 weeks (167 women) and 34 weeks (160 women) with cord blood from 93 infants. Results from untargeted metabolite analysis (ultrahigh performance LC-MS) are presented as adjusted logistic/linear regression of maternal and cord blood metabolites, risk factors and perinatal complications using a modified Bonferroni limit of significance for dependent variables.

**Results:**

Maternal continuous glucose monitoring time-above-range (but not BMI or excessive gestational weight gain) was associated with increased triacylglycerols in maternal blood and increased carnitines in cord blood. LGA, adiposity, neonatal hypoglycaemia and offspring hyperinsulinism showed distinct metabolite profiles. LGA was associated with increased carnitines, steroid hormones and lipid metabolites, predominantly in the third trimester. However, neonatal hypoglycaemia and offspring hyperinsulinism were both associated with metabolite changes from the first trimester, featuring triacylglycerols or dietary phenols. Pre-eclampsia was associated with increased abundance of phosphatidylethanolamines, a membrane phospholipid, at 24 weeks.

**Conclusions/interpretation:**

Altered lipid metabolism is a key pathophysiological feature of type 1 diabetes pregnancy. New strategies for optimising maternal diet and insulin dosing from the first trimester are needed to improve pregnancy outcomes in type 1 diabetes.

**Graphical Abstract:**

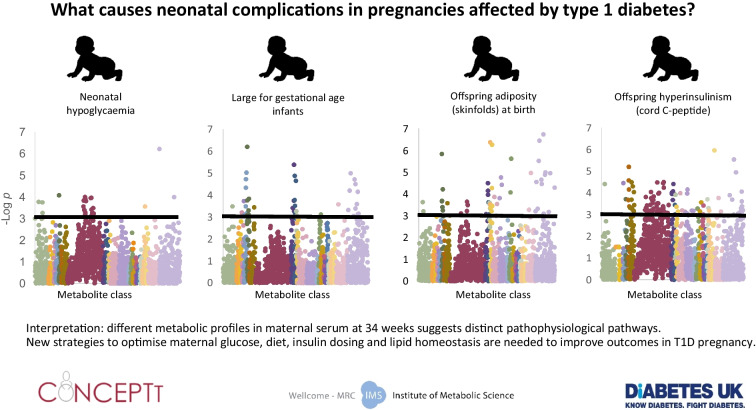

**Supplementary Information:**

The online version of this article (10.1007/s00125-023-05989-2) contains peer-reviewed but unedited supplementary material.


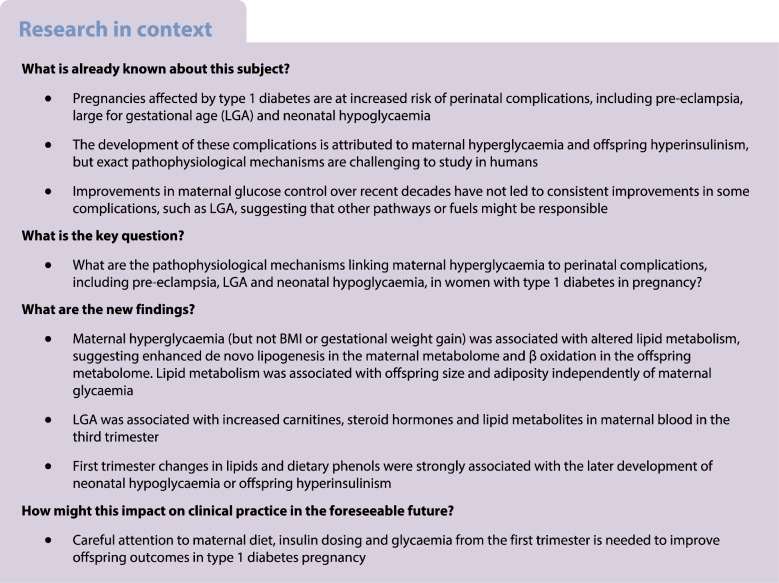


## Introduction

Type 1 diabetes in pregnancy is associated with perinatal complications including pre-eclampsia, large for gestational age (LGA) and neonatal hypoglycaemia, attributed to sustained maternal hyperglycaemia [1]. However, despite improvements in glycaemic control, pregnancy outcomes remain suboptimal in type 1 diabetes compared with healthy pregnancy [2, 3]. For example, 60% of infants were LGA in the Continuous Glucose Monitoring in Women with Type 1 Diabetes in Pregnancy Trial (CONCEPTT) despite improved antenatal glucose control [3]. This has led to speculation that other metabolic processes or fuels may also contribute to perinatal complications [1, 4].

LGA is likely to involve altered carbohydrate, protein and fat metabolism and is influenced by multiple factors, including maternal obesity [5], gestational weight gain [5], dietary quality [6], maternal lipids [7] and glycaemia [8, 9]. Neonatal hypoglycaemia, a disorder of carbohydrate metabolism affecting 25% of offspring [3], is associated with fetal hyperinsulinism and has been attributed to suboptimal maternal glycaemia during late gestation and birth [10], but may occur earlier in pregnancy than previously recognised [8, 11, 12]. Compared to healthy women, mothers with type 1 diabetes in pregnancy have a five times increased risk of pre-eclampsia [13] which can be predicted using first trimester biomarkers, including leptin and glucose [14], and protein-related biomarkers at 28 weeks [15]. The relative contribution of carbohydrate, lipid and protein metabolism to the development of pre-eclampsia in type 1 diabetes pregnancy are unclear.

Using samples from the CONCEPTT RCT, we assessed metabolomic and lipidomic changes associated with modifiable risk factors for suboptimal outcomes (hyperglycaemia, maternal BMI, gestational weight gain, habitual diet) and specific pregnancy complications, including LGA and offspring adiposity, neonatal hypoglycaemia, offspring hyperinsulinism and pre-eclampsia in pregnancies affected by type 1 diabetes.

## Methods

### Patients

Pregnant women with type 1 diabetes were recruited into the CONCEPTT trial, described more fully elsewhere [3] (ClinicalTrials.gov NCT01788527; trial registered 11/2/2013). In brief, 225 women with type 1 diabetes were recruited in early pregnancy or pre-pregnancy and completed the study, of whom 200 had a liveborn singleton infant. Women with type 1 diabetes who were pregnant or planning pregnancy were recruited from 31 hospitals in Canada, England, Scotland, Spain, Italy, Ireland and the USA. Most women were of European ethnicity (self-reported; 86.2%) and participants had a mean (SD) age of 31.4 (4.5) years and BMI of 25.8 (4.6) kg/m^2^ with diabetes duration of 16.5 (7.7) years. Most women had education post-secondary school (>75%) and many used an insulin pump (48.9%). A subset of these participants (174/200) gave at least one additional non-fasting serum sample for the biorepository at 12 weeks (147 women), 24 weeks (167 women) and 34 weeks (160 women) with additional samples of cord blood (93 infants) for metabolomics analysis. This subset was similar to the overall CONCEPTT population (Table 1). Samples were rapidly processed and stored frozen at −80°C prior to batch analysis. Adjudicated outcome definitions were used [3] for pre-eclampsia (blood pressure ≥140/90 mmHg with proteinuria [urine ≥1+ protein] on >1 occasion); neonatal hypoglycaemia (defined as necessitating intravenous dextrose at ≤48 h of life) and LGA (birthweight >97.7th centile using customised Gestation Related Optimal Weight (GROW) centiles, calculated using version 8 [2017] of the GROW calculator [16]). All study participants gave written informed consent. The study was approved by the research ethics committee (12/EE/0310) and was carried out in accordance with the Declaration of Helsinki (2013).

**Table 1:** Baseline participant characteristics

Characteristic	All CONCEPTT participants with livebirth and neonatal outcome data available *n*=225	All participants who gave at least one additional sample for metabolomics measurement *n*=174
Demographic details		
Maternal age, years	31.44 (4.5)	31.44 (4.7)
Pre-pregnancy BMI, kg/m^2^	25.80 (4.6)	25.60 (4.4)
European ethnicity %	194/225 (86.2)	151/174 (86.8)
Primiparous %	114/225 (50.7)	83/174 (47.7)
Diabetes history		
Insulin pump %	110/225 (48.9)	87/174 (50.0)
Intervention (CGM) %	110/225 (48.9)	82/174 (47.1)
Duration T1DM, years	16.53 (7.7)	16.38 (7.8)
Age of onset of T1DM, years	14.91 (8.0)	15.06 (8.2)
Glycaemia at 12 weeks		
HbA_1c_, mmol/mol	51.76 (6.6)	51.42 (6.5)
HbA_1c_, %	6.89 (0.6)	6.85 (0.6)
%TIR, 3.5–7.8 mmol/l	51.71 (13.1)	51.48 (12.6)
% TAR, >7.8 mmol/l	39.75 (14.3)	39.98 (14.0)
% Time below range, <3.5 mmol/l	8.53 (7.1)	8.54 (6.8)
Glycaemia at 24 weeks		
HbA_1c_, mmol/mol	45.57 (6.8)	45.48 (6.9)
HbA_1c_, %	6.32 (0.6)	6.31 (0.6)
%TIR, 3.5–7.8 mmol/l	51.14 (15.2)	51.00 (15.5)
% TAR, >7.8 mmol/l	43.68 (16.9)	43.60 (17.0)
% Time below range, <3.5 mmol/l	5.16 (5.3)	5.40 (5.5)
Glycaemia at 34 weeks		
HbA_1c_, mmol/mol	46.67 (6.8)	46.71 (6.9)
HbA_1c_, %	6.42 (0.6)	6.42 (0.6)
%TIR, 3.5–7.8 mmol/l	64.35 (14.7)	64.10 (14.9)
% TAR, >7.8 mmol/l	30.58 (14.9)	30.94 (14.9)
% Time below range, <3.5 mmol/l	5.11 (5.0)	4.95 (4.7)
Pregnancy outcomes		
Pre-eclampsia %	27/225 (12.0)	20/174 (11.5)
Neonatal sex, % male	110/225 (48.9)	84/174 (48.3)
Gestational age at birth, weeks	36.99 (1.6)	37.04 (1.6)
Pre-term birth %	89/225 (39.6)	70/174 (40.3)
Caesarean section %	155/225 (68.9)	116/174 (66.7)
LGA, >97.7th centile %	94/225 (41.8)	68/174 (39.1)
Respiratory distress %	19/225 (8.4)	12/174 (6.9)
Neonatal hypoglycaemia %	57/225 (25.3)	47/174 (27.0)
NICU admission %	83/225 (36.9)	59/174 (33.9)
Hyperbilirubinaemia %	62/225 (27.6)	42/174 (24.1)
Cord blood C-peptide, pmol/l	1110.2 (1063.8)	1110.2 (1063.8) *n*=93

Data shown as mean (SD) or *n* (%)

NICU, neonatal intensive care unit; T1DM, type 1 diabetes mellitus

### Laboratory methods

Untargeted metabolite profiling was performed by Metabolon (Munich, Germany) using a Waters ACQUITY ultrahigh performance LC (UPLC; Elstree, UK) and Thermo Scientific Q-Exactive high-resolution MS with a heated electrospray ionisation (HESI-II) source (Waltham, USA). Two aliquots were analysed using acidic positive ion conditions, which included optimisation step for accurate determination of hydrophilic or hydrophobic compounds. Two further aliquots were analysed using negative ionisation mode. The MS analysis alternated between full scan MS (scan range 70–1000 m/z, scan rate 4 Hz) and data-dependent MS^n^ scans using dynamic exclusion.

Compounds were identified using retention index, mass match ±10 ppm, and MS/MS forward and reverse scores between the sample data and the authenticated standards using a library, maintained by Metabolon. Peaks were quantified using AUC; raw data were normalised to correct for instrument inter-day tuning differences (median 1.0 for each run).

Lipids were analysed using the Metabolon complex lipid panel. Lipids were extracted using a modified Bligh-Dyer extraction (methanol/water/dichloromethane) with ^2^H-labelled internal standards. The extracts were dried under nitrogen and reconstituted with ammonium acetate in dichloromethane:methanol. The extracts were transferred to vials for infusion-MS, performed on a Shimadzu LC with nano PEEK tubing and the Sciex SelexION-5500 QTRAP. Samples were analysed via both positive and negative mode electrospray. The 5500 QTRAP was operated in multiple reaction monitoring mode. Individual lipid species were quantified by taking the ratio of the signal intensity of each target compound to that of its assigned internal standard, then multiplying by the concentration of internal standard added to the sample. Several different lipid classes were profiled, including cholesteryl esters, NEFAs, monoacylglycerols, diacylglycerols (DAGs), triacylglycerols (TAGs), phosphatidylcholines (PCs), phosphatidylethanolamines (PEs), phosphatidylinositols (PIs) and lysophosphatidylcholines (LPCs). Lipid class concentrations were calculated from the sum of all molecular species assigned to that class. Fatty acid compositions were determined by calculating the proportion of each class comprised by individual fatty acids.

### Statistical analysis

Data are presented as mean (SD) or *n* (%) as appropriate. Analysis was performed independently by two authors (CLM and AK). Principal component analysis and sparse partial least squares-discriminant analysis were carried out to identify outliers and to identify preliminary variables which differentiated the groups, using a Student’s *t* test. Logistic regression analysis was performed to assess the importance of covariates to the outcomes. To enable comparison between analytes of different abundance, unadjusted metabolomics variables were standardised before regression. For logistic regression (categorical outcomes), results are presented as standardised ORs and 95% CIs, i.e. showing the change in the metabolite (units: SDs) associated with the outcome. For linear regression (continuous outcome variables), results are presented as standardised coefficients (Coeff) and 95% CI, i.e. showing the change in the metabolite (units: SDs) associated with a one unit change in the outcome (for example, 1% increase in maternal time-above-range [TAR], 1 pmol/l increase in cord blood C-peptide).

Three models were presented for each outcome for maternal and cord blood analyses. For maternal analyses, Model 1 was unadjusted and Model 2 was adjusted for maternal age, pre-pregnancy BMI, ethnicity (European/non-European), parity (primiparous/multiparous), highest educational qualification and neonatal sex. Model 3 was adjusted for all factors in Model 2, but was also adjusted for concurrent maternal glycaemia, measured using CGM % time-in-range (%TIR; 3.5–7.8 mmol/l). We performed a preliminary assessment of the effect of the trial arm upon metabolites, independently of glycaemia, with no statistically significant results. We therefore did not adjust the analyses for the trial arm. For cord blood analyses, Model 1 was unadjusted and Model 2 was adjusted for maternal age, pre-pregnancy BMI, ethnicity (European/non-European), parity (primiparous/multiparous), highest educational qualification, neonatal sex, gestational age at birth, Caesarean delivery and antenatal steroid use. Model 3 was adjusted for all factors in Model 2, but also included maternal glycaemia at 34 weeks, measured using CGM %TIR (3.5–7.8 mmol/l). Stata 16.0 (StataCorp, College Station, TX, USA) was used for all analyses.

To take account of the large number of inter-related variables, a modified Bonferroni method [17] was made using *p*=0.05/(square root of *n*), to allow adjustment for multiple testing. This resulted in a threshold of significance of *p*<0.0011. For comparison, we have also included analysis based on a Benjamini–Hochberg false discovery rate correction (*q*<0.05) in the supplementary material. A subset analysis, assessing the effect of a range of pre-specified carnitine and fatty acids species (*n*=36) upon offspring size and skinfold thickness used *p*=0.01 (*α*=1%) as the limit of significance (*p*=0.05 /[square root of *n*]).

Our sample size (*n*=174) provides >90% power to detect differences in metabolite abundance of 0.5 SDs for LGA, 0.7 SDs for neonatal hypoglycaemia and 1.0 SD for pre-eclampsia, with higher power for continuous outcome variables (e.g. hyperglycaemia, BMI). For cord blood, our sample size (*n*=93) provides >90% power to detect differences in metabolite abundance of 0.75 SDs. As participants who did not provide a voluntary sample for metabolomics analysis were not included in this analysis, there were few missing data points which were missing at random (no imputation). Analyses where an analyte was not detected were considered to have undetected analyte (i.e. zero), and were not considered to have missing data. Metabolomic variables were only included if they were identified in at least 20 samples.

## Results

Participant characteristics (Table 1) were similar to the original cohort. Associations of maternal and offspring metabolites with maternal BMI, gestational weight gain and maternal diet at 12 weeks are provided in Appendix 2 (electronic supplementary material [ESM] Figs A2.1–2.3; ESM Tables 1–8) [18]. Coefficients (Coeff)/ORs and confidence intervals are provided for the strongest associations only.

### Metabolomic and lipidomic changes associated with maternal hyperglycaemia

**Fig. 1:**
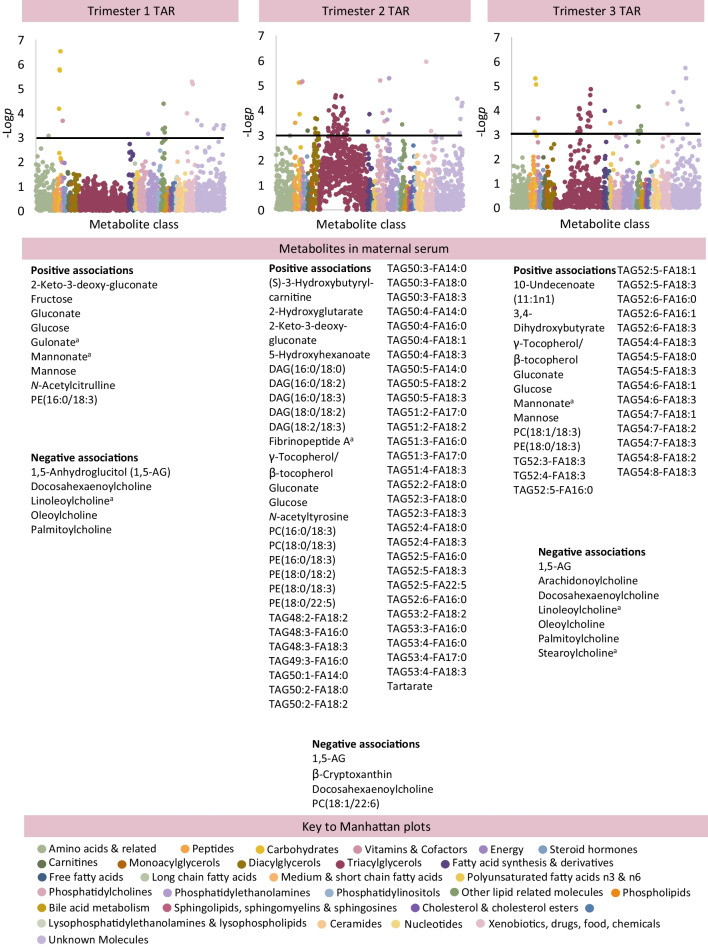
Associations between individual metabolites in maternal blood in association with maternal hyperglycaemia in trimesters 1, 2 and 3, measured as TAR on CGM. For analysis of the maternal metabolome, the graphs show results adjusted for maternal age, pre-pregnancy BMI, ethnicity (European/non-European), parity (primiparous/multiparous), highest educational qualification and neonatal sex (Model 2). Only metabolites meeting *p*<0.0011 (−log*p*>3, above black line) are considered statistically significant. Unknown metabolites are included in the graphs but not listed in these figures (available in ESM tables). The key for the Manhattan plots shown in Fig. 1 applies to all figures. ^a^There is some uncertainty about the exact chemical identity of the mass spectrometry peak for this species; the species listed is considered the most likely option where a range of possibilities exist. FA, fatty acid

Maternal TAR was associated with multiple changes in the maternal metabolome on adjusted analysis (Fig. 1), ESM Table 9) [18]. Maternal TAR at 12 weeks was negatively associated with 1,5-anhydroglucitol (1,5-AG) (Coeff [95% CI] −2.60 [−3.85, −1.35]) and linoleoylcholine (Coeff −1.84 [−2.70, −0.98]), and positively associated with several monosaccharides (for example, glucose [Coeff 3.04 (1.84, 4.25)], fructose, mannose, and derivatives gluconate, 2-keto-3-deoxy-gluconate, mannonate) and gulonate.

Maternal TAR at 24 weeks was negatively associated with 1,5-AG (Coeff −1.94 [−2.76, −1.11]), docosahexaenoylcholine, β-cryptoxanthin (Coeff −2.09 [−2.98, −1.20]), and PC(18:1/22:6) (Coeff −1.61 [−2.47, −0.76]). There were positive associations with monosaccharide derivatives, tartarate (Coeff 2.47 [1.02, 3.92]), with 2-hydroxyglutarate (structurally related to ketoglutarate [tricarboxylic acid cycle]), and fibrinopeptide A, γ-tocopherol/β-tocopherol and *N*-acetyltyrosine. The most statistically significant positive associations at 24 weeks involved lipid metabolism, including a carnitine (β oxidation; Coeff 1.15 [0.50, 1.80]), several individual DAGs, 36 individual TAGs (containing 48–53 carbons and 0–6 double bonds; fatty acids 16:0, 17:0, 18:0, 18:2 and 18:3 were abundant) and several PCs and PEs. Maternal obesity or high-fat diet did not yield comparable increases in maternal serum TAGs (ESM Appendix 2).

Maternal TAR at 34 weeks showed negative associations with six choline species (for example, docosahexaenoylcholine, Coeff −2.14 [−3.17, −1.11]) and 1,5-AG (Coeff −2.08 [−3.28, −0.89]). There were positive associations with monosaccharides and derivatives (for example, glucose Coeff 2.68 [1.57, 3.79]), 10-undecenoate, 3,4-dihydroxybutyrate, γ-tocopherol/β-tocopherol, PC(18:1/18:3), PE(18:0/18:3) and 18 individual TAGs (containing 52–54 carbons and 0–3 double bonds; NEFAs 16:0, 16:1, 18:0, 18:1, 18:2 and 18:3 were prominent).

**Fig. 2:**
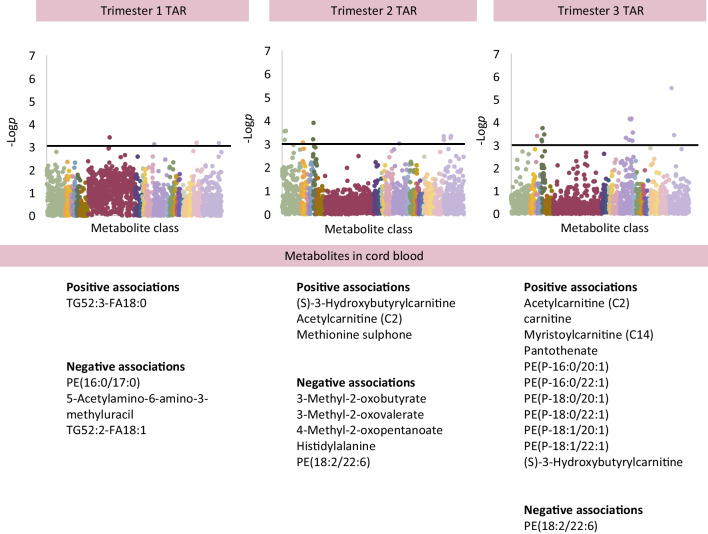
Associations between individual metabolites in cord blood in association with maternal hyperglycaemia in trimesters 1, 2 and 3, measured as TAR on CGM. For analysis of the cord blood metabolome, the graphs show results adjusted for maternal age, pre-pregnancy BMI, ethnicity (European/non-European), parity (primiparous/multiparous), highest educational qualification, neonatal sex, gestational age at birth, Caesarean delivery and antenatal steroid use (Model 2). Only metabolites meeting *p*<0.0011 (−log*p*>3, above black line) are considered statistically significant. Unknown metabolites are included in the graphs but not listed in these figures (available in ESM tables)

Maternal TAR at 12, 24 and 34 weeks demonstrated associations with cord blood metabolites (Fig. 2; adjusted analysis). Maternal TAR at 12 weeks demonstrated negative associations with PE(16:0/17:0), 5-acetylamino-6-amino-3-methyluracil (Coeff −3.04 [−4.74, −1.35]) and a TAG (TAG52:2 containing FA18:1) and a positive association with another TAG (TAG52:3 containing FA18:0; Coeff 2.86 [1.33, 4.39]). Maternal TAR at 24 weeks was positively associated with carnitines (for example, acetylcarnitine, Coeff 2.31 [1.18, 3.45]), and methionine sulphone. There were negative associations with 3-methyl-2-oxobutyrate, 3-methyl-2-oxovalerate, 4-methyl-2-oxopentanoate, histidylalanine and PE(18:2/22:6). Maternal TAR at 34 weeks was negatively associated with PE(18:2/22:6) and was positively associated with carnitines, six PE plasmalogens (for example, PE[P-18:0/22:1], Coeff 3.13 [1.66, 4.59]), and pantothenate.

### Metabolomic and lipidomic changes associated with LGA

**Fig. 3:**
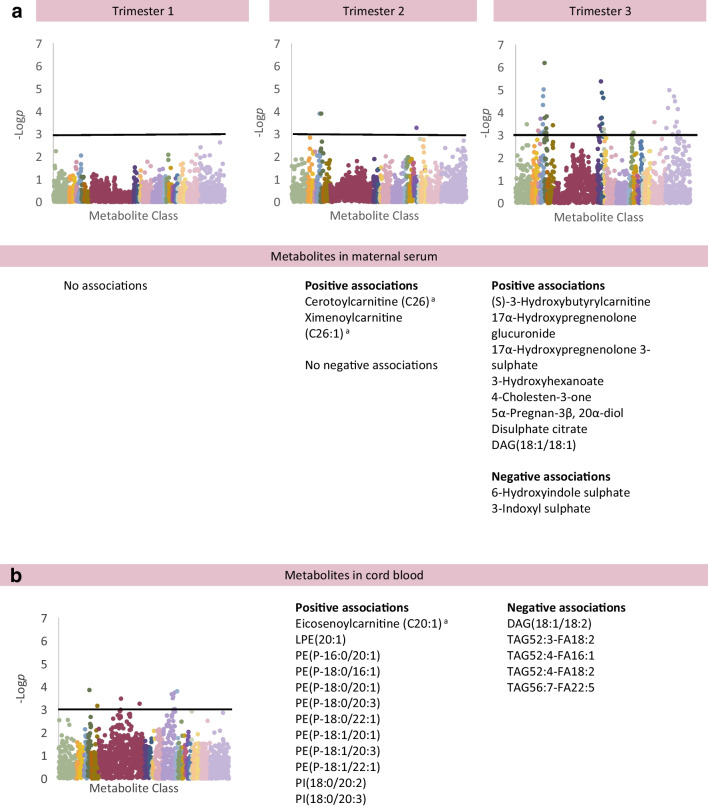
Associations between individual metabolites in maternal blood in trimesters 1, 2 and 3 (**a**) and in cord blood (**b**) in association with LGA at birth. For analysis of the maternal metabolome, the graphs show results adjusted for maternal age, pre-pregnancy BMI, ethnicity (European/non-European), parity (primiparous/multiparous), highest educational qualification, neonatal sex and maternal TIR on CGM at the appropriate timepoint (Model 3). For analysis of the cord blood metabolome, the graphs show results adjusted for maternal age, pre-pregnancy BMI, ethnicity (European/non-European), parity (primiparous/multiparous), highest educational qualification, neonatal sex, gestational age at birth, Caesarean delivery, antenatal steroid use and maternal TIR on CGM at 34 gestational weeks (Model 3). Only metabolites meeting *p*<0.0011 (−log*p*>3, above black line) are considered statistically significant. Unknown metabolites are included in the graphs but not listed in these figures (available in ESM tables). ^a^There is some uncertainty about the exact chemical identity of the mass spectrometry peak for this species; the species listed is considered the most likely option where a range of possibilities exist. LPE, lysophosphatidylethanolamine

LGA was associated with 12 metabolites in cord blood and 12 variables in maternal blood (Fig. 3, ESM Table 10) [18]. At 12 weeks, there were no statistically significant metabolite associations with LGA on any model. In maternal blood at 24 weeks, cerotoylcarnitine (Coeff 2.49 [1.53, 4.07]) and ximenoylcarnitine showed positive associations with subsequent development of LGA. In maternal blood at 34 weeks, (S)-3-hydroxybutyrylcarnitine (OR [95% CI] 2.66 [1.56–4.54]), and steroid hormones (17-α-hydroxypregnenolone 3-sulphate, OR 2.88 [1.63, 5.07]) continued to show positive associations with LGA. A fatty acid derivative [3-hydroxyhexanoate] and citrate (TCA cycle) were also positively associated with LGA. Two metabolites were negatively associated with LGA (6-hydroxyindole sulphate, 3-indoxyl sulphate).

In cord blood (Fig. 3), adjusted analyses demonstrated profound negative associations between LGA and several polyunsaturated TAGs (C52–56; 3–7 double bonds; strongest TAG[52:4], OR 0.12 [0.04, 0.38]) and an unsaturated DAG (DAG[18:1/18:2]). Positive associations were identified with eicosenoylcarnitine (OR 7.64 [2.68, 21.76]), two phosphoinositols (for example, PI[18:0/20:3], OR 10.74 [3.14, 36.82]), a lypophosphoethanolamine and multiple PE plasmalogens (for example, PE[P-18:1/20:1], OR 13.74 [3.49, 54.14]).

### Metabolomic and lipidomic changes associated with offspring adiposity

We assessed the effects of maternal and cord blood metabolites upon offspring adiposity, as evidenced by skinfold sum (ESM Appendix 3; ESM Figs A3.1–3.3; ESM Table 11) [18]. On adjusted analysis, multiple metabolites, including carnitine species, plasmalogens and TAGs in maternal or cord blood were positively associated with skinfold sum independently of maternal HbA_1c_ at 34 weeks (ESM Appendix 3; ESM Fig. A3.1). The strongest associations in maternal blood were at 34 weeks including glycerol (Coeff per 1 mm increase in skinfold sum 0.09 [0.06, 0.13]), PE(18:0/18:3) (Coeff 0.09 [0.05, 0.13]), fatty acid derivatives (such as 10-undecenoate [Coeff 0.08 (0.05, 0.11)]) and several carnitines (e.g. (S)-3-hydroxybutyrylcarnitine, Coeff 0.08 [0.05, 1.10]). In cord blood, the strongest associations were with salicylate (Coeff 0.11 [0.06, 0.16]) and salicylurate (Coeff 0.11 [0.06, 0.16]), adenosine 5'-diphosphoribose (ADP-ribose) (Coeff 0.10 [0.06, 0.15]), multiple TAGs (e.g. TAG[56:7], Coeff 0.10 [0.05, 0.15]) and margaroylcarnitine (Coeff 0.10 [0.05, 0.14]). A mediation analysis demonstrated that lipid metabolites were likely to be important mediators of the relationship between maternal hyperglycaemia and offspring adiposity, but did not solely mediate this relationship (ESM Appendix 3; ESM Figs A3.2–3.3).

### Metabolomic and lipidomic changes associated with neonatal hypoglycaemia

**Fig. 4:**
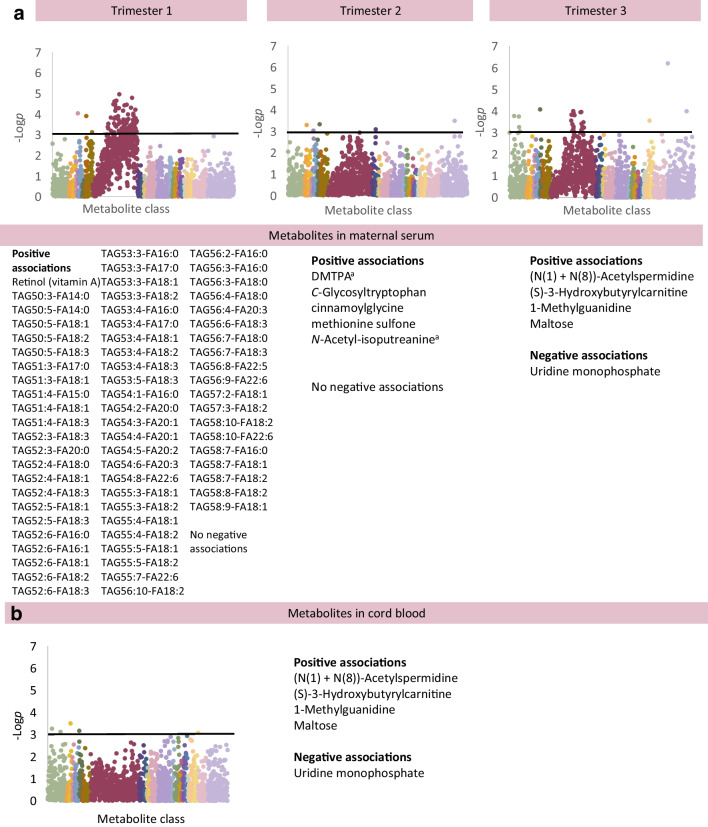
Associations between individual metabolites in maternal blood in trimesters 1, 2 and 3 (**a**), and in cord blood (**b**) in association with neonatal hypoglycaemia (NH). For analysis of the maternal metabolome, the graphs show results adjusted for maternal age, pre-pregnancy BMI, ethnicity (European/non-European), parity (primiparous/multiparous), highest educational qualification, neonatal sex and maternal TIR on CGM at the appropriate timepoint (Model 3). For analysis of the cord blood metabolome, the graphs show results adjusted for maternal age, pre-pregnancy BMI, ethnicity (European/non-European), parity (primiparous/multiparous), highest educational qualification, neonatal sex, gestational age at birth, Caesarean delivery, antenatal steroid use and maternal TIR on CGM at 34 gestational weeks (Model 3). Only metabolites meeting *p*<0.0011 (−log*p*>3, above black line) are considered statistically significant. Unknown metabolites are included in the graphs but not listed in these figures (available in ESM tables). ^a^There is some uncertainty about the exact chemical identity of the mass spectrometry peak for this species; the species listed is considered the most likely option where a range of possibilities exist. DMTPA, 2,3-dihydroxy-5-methylthio-4-pentenoate; FA, fatty acid

Neonatal hypoglycaemia was associated with five variables in cord blood and 72 variables in maternal blood (Fig. 4; ESM Table 12) [18]. In maternal blood at 12 weeks, multiple individual TAGs were positively associated with neonatal hypoglycaemia, particularly polyunsaturated isoforms (3–9 double bonds). The strongest association was seen with TAG(56:2) at 12 weeks (OR 7.93 [2.70, 23.33]). Retinol, a fat-soluble vitamin, was also positively associated with neonatal hypoglycaemia at 12 weeks (OR 2.48 [1.49, 4.13]). There were no statistically significant associations between total TAGs or retinol content at 12 weeks and HbA_1c_ or CGM %TIR at any timepoint. Standardised logistic regression analysis confirmed a positive association between total TAGs at 12 weeks and future development of neonatal hypoglycaemia (OR 3.95 [1.69–9.22]).

At 24 weeks, no species were associated with subsequent neonatal hypoglycaemia in offspring. At 34 weeks, derivatives of a fatty acid, amino acids and a polyamine (2,3-dihydroxy-5-methylthio-4-pentenoate [DMTPA] [OR 2.27 (1.44, 3.56)], *C*-glycosyltryptophan, cinnamoylglycine, methionine sulphone, *N*-acetyl-isoputreanine) were positively associated with subsequent neonatal hypoglycaemia.

In cord blood, uridine monophosphate (OR 0.10 [0.09, 0.53]) was negatively associated with neonatal hypoglycaemia. Positive associations were seen with maltose, (S)-3-hydroxybutyrylcarnitine (OR 4.09 [1.81, 9.23], a polyamine ((N(1) + N(8))-acetylspermidine, OR 9.59 [2.66, 34.60]) and methylguanidine (OR 9.59 [2.66, 34.60]).

### Metabolomic and lipidomic changes associated with cord C-peptide

**Fig. 5:**
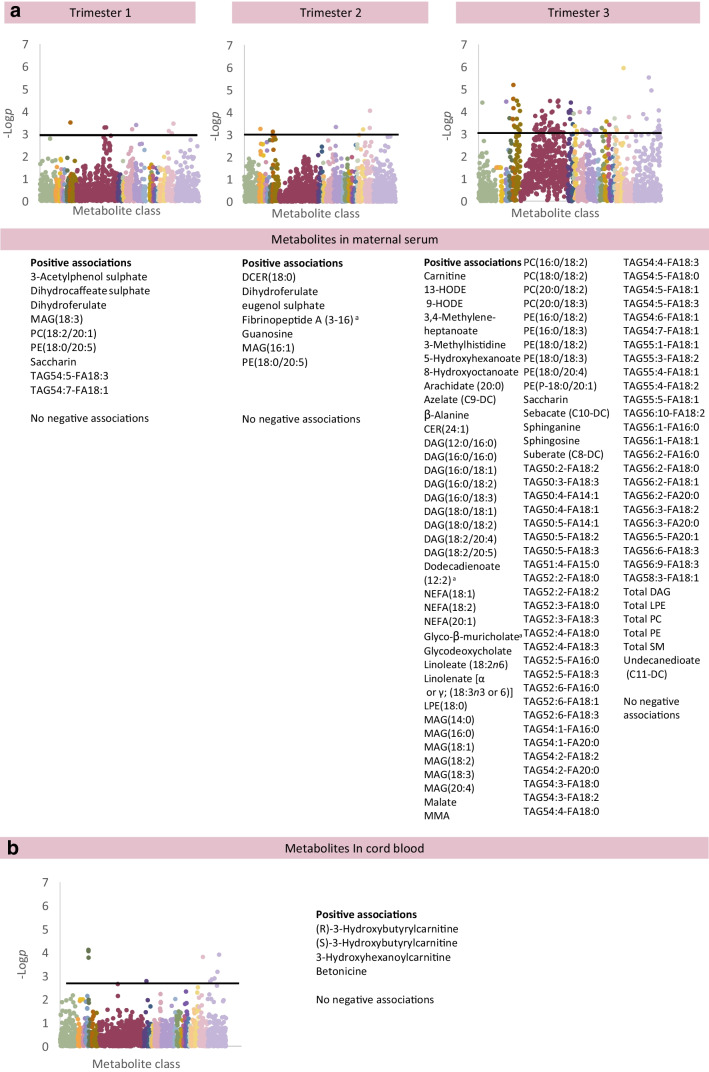
Associations between individual metabolites in maternal blood in trimesters 1, 2 and 3 (**a**), and in cord blood (**b**) in association with cord blood C-peptide. For analysis of the maternal metabolome, the graphs show results adjusted for maternal age, pre-pregnancy BMI, ethnicity (European/non-European), parity (primiparous/multiparous), highest educational qualification, neonatal sex and maternal TIR on CGM at the appropriate timepoint (Model 3). For analysis of the cord blood metabolome, the graphs show results adjusted for maternal age, pre-pregnancy BMI, ethnicity (European/non-European), parity (primiparous/multiparous), highest educational qualification, neonatal sex, gestational age at birth, Caesarean delivery, antenatal steroid use and maternal TIR on CGM at 34 gestational weeks (Model 3). Only metabolites meeting *p*<0.0011 (−log*p*>3, above black line) are considered statistically significant. Unknown metabolites are included in the graphs but not listed in these figures (available in ESM tables). ^a^There is some uncertainty about the exact chemical identity of the mass spectrometry peak for this species; the species listed is considered the most likely option where a range of possibilities exist. CER, ceramide; DCER, dihydroceramide; FA, fatty acid; HODE, hydroxyoctadecadienoic acid; LPE, lysophosphatidylethanolamine; MAG, monoacylglycerol; MMA, (S)-3-hydroxybutyryl-methylmalonate; SM, sphingomyelin

Neonatal hyperinsulinism (cord blood C-peptide) was associated with metabolite changes in maternal blood from the first trimester (Fig. 5; ESM Table 13) [18]. At 12 weeks, several phenols (including saccharin, Coeff per 1 pmol increase in C-peptide 0.0011 [0.0005, 0.0017]) and lipids (monoglyceride, PC, PE and two TAGs) showed positive associations. At 24 weeks, two phenols (strongest dihydroferulate, Coeff 0.0004 [0.0002, 0.0007]), several lipids, fibrinopeptide A (3–16) and guanosine showed positive associations. In maternal serum at 34 weeks, 107 species showed positive associations with cord C-peptide including a phenol (saccharin), energy-related species (malate; methylmalonate) and many different lipid species, such as fatty acids and derivatives (hydroxyhexanoate, hydroxyoctanoate, suberate, sebacate, undecanedioate, dodecadienoate), and multiple mono-, di- and triacylglycerols. There were positive associations with polyunsaturated fatty acids (PUFAs; linolenate and linoleate) and molecules which incorporate PUFAs into phospholipids (e.g. 13- or 9-hydroxyoctadecadienoic acid [HODE]) and multiple phospholipid species (PCs, PEs, plasmalogens). The cord blood metabolome had several statistically significant positive associations with cord C-peptide, including three carnitines and betonicine (Coeff 0.0008 [0.0004, 0.0012]).

### Metabolomic and lipidomic changes associated with pre-eclampsia

Out of 174 women who gave samples for metabolite profiling, 20 developed pre-eclampsia, of whom 12 had cord blood available (ESM Appendix 4; Fig. A4.1; ESM Table 14) [18]. Pre-eclampsia was associated with 21 variables in maternal blood (ESM Appendix 4; Fig. A4.2). No variables in cord blood were associated with pre-eclampsia, perhaps owing to lower statistical power in this group (ESM Appendix 4; Fig. A4.1).

In maternal blood at 12 weeks, two TAGs were positively associated with pre-eclampsia. At 24 weeks, N-acetylhistidine, orotidine and nine individual PEs (containing FA[16:0], FA[18:0) or FA[18:1]) in maternal blood were associated with pre-eclampsia. At 34 weeks, there were positive associations between pre-eclampsia development and several amino acid derivatives, polyamines and an endogenous or exogenous pentose alcohol (arabitol or xylitol).

## Discussion

Type 1 diabetes in pregnancy is associated with altered carbohydrate, lipid and protein metabolism from the first trimester, which strongly predict perinatal complications. Inadequate availability of insulin, a master regulator of carbohydrate, lipid and protein metabolism, is a likely unifying cause. Maternal hyperglycaemia was associated with increased lipid abundance in maternal blood and increased carnitines in cord blood, changes which were associated with offspring adiposity independently of maternal glucose. Neonatal hypoglycaemia and offspring hyperinsulinism were both associated with first trimester changes in metabolites related to maternal lipid metabolism and dietary intake of macronutrients and phenols.

### Strengths of the study

CONCEPTT was a large, multicentre, multinational RCT with extensive CGM data, LGA stringently defined using a customised centile threshold of >97.7th percentile for these analyses and predefined clinical endpoints, including pre-eclampsia and neonatal hypoglycaemia. Statistical analysis was performed independently by two co-authors, blinded to metabolite names using three different logistic regression models. Paired cord blood specimens in 93 pregnancies allows comparison of maternal–fetal metabolism.

### Limitations of the study

Maternal blood was not collected in the fasting state, which increases biological variability for some metabolites. Data on diet (*n*=56 at 12 weeks), gestational weight gain (*n*=141), cord blood (*n*=93) and samples from women with pre-eclampsia (20/174) were only available on a subset, leading to reduced statistical power for these analyses.

MS is a powerful tool, but the metabolites identified provide only a snapshot of metabolism at key timepoints in the life of mother and child. Cord blood was taken immediately after delivery, a highly dynamic time in metabolism which is difficult to study in humans with clarity. While we adjusted for key perinatal exposures, such as gestational age at delivery, maternal glycaemia and Caesarean section, we cannot exclude the possibility of other influences, which we could not resolve statistically. Despite these limitations, the cord blood results provide intriguing data about early life, and are useful for generating new hypotheses.

A further limitation is that insulin, the master regulator of carbohydrate, lipid and protein metabolism, was not directly measured in this study. Inadequate insulin supply, through inadequate dosing for maternal diet or insulin resistance, results in insufficient carbohydrate availability for cellular metabolism, provoking mobilisation of lipid and protein stores to meet fuel requirements.

### Main findings

#### Hyperglycaemia

Maternal hyperglycaemia was associated with increased lipid abundance in maternal blood and increased carnitines in cord blood. There are several main sources of fatty acids in maternal blood: (1) dietary intake; (2) lipogenesis; and (3) lipolysis, which are all dynamically regulated by human pregnancy but produce a different fatty acid profile in serum. Dietary data suggest that serum lipid abundance is not related to a maternal high-fat diet (ESM Tables 7–8). Lipolysis is typically active in late pregnancy due to reduced lipoprotein lipase activity in parallel with rising insulin resistance [19]. The fatty acid profile in maternal blood showed an increased abundance of species related to de novo lipogenesis (containing fatty acids 16:0, 16:1, 18:0 or 18:1; for example in DAG[32:0] or TAG[48:0]). Lipogenesis-related species have been associated with hyperglycaemia in other populations [20, 21]. As de novo lipogenesis is a physiological pathway associated with using up excess glucose, it seems highly likely that it is active in diabetes in pregnancy.

Carnitines were abundant in cord blood in association with maternal hyperglycaemia and adiposity. Carnitines are essential cofactors required for the transportation of long-chain fatty acids into the mitochondrion for β oxidation, the process of generating ATP from fatty acids, typically active in states of low glucose availability. This is a surprising finding, as our current understanding of early human metabolism suggests that the fetus is entirely reliant on glycolysis as a means of fuel production, with β oxidation ‘switched on’ after birth, once the infant metabolism has to adjust to fasting. Inherited or acquired defects in fatty acid oxidation cause increases in the abundance of carnitines and present from day 3 of life, not from birth (for example, C4-C18:1 acylcarnitines in multiple acyl-CoA dehydrogenase deficiency [MADD]) [22]. Testing carnitines in cord blood does not reliably identify these conditions owing to insufficient duration of fasting [23, 24]. Carnitines in cord blood may reflect maternal physiology [23, 24] but there was no evidence of widespread changes in maternal carnitines as a result of hyperglycaemia. We therefore consider that elevated carnitines in cord blood are more likely to signify an unexpected increase in β oxidation in the fetus before birth. While the fetus is known to rely exclusively on glycolysis to meet its fuel needs in a healthy pregnancy, very few previous studies have addressed fetal fuel utilisation in pregnancies affected by diabetes.

Adiposity in offspring was associated with carnitines and abundant lipid species with lipogenesis-related fatty acids. β oxidation and de novo lipogenesis do not usually run concurrently but can do so, for example, as described in adults with obesity or after over-feeding, where an energy-consuming cycle of β oxidation and de novo lipogenesis is likely to reduce excess fuel from excess calories [25, 26]. A comparable situation is possible in the fetus and may dissipate the excess fuel/energy received from the maternal circulation in addition to providing basic substrates for fetal growth (e.g. phospholipids).

#### Offspring size and adiposity

LGA was not associated with first trimester metabolomic changes in maternal blood, but by 24 weeks, a pattern had emerged featuring multiple metabolites with a role in promoting or sustaining excessive fetal growth (Fig. 3). There was evidence of increased steroid hormone production, and increased activity of the TCA cycle (citrate) and β oxidation pathway (carnitines) suggesting abundant energy production. LGA and neonatal adiposity, measured as the sum of four skinfolds, showed strikingly different metabolite profiles, suggesting that increased size overall (LGA) and increased adiposity are not completely synonymous conditions in the context of type 1 diabetes pregnancy. Our mediation analysis suggested that disordered lipid metabolism may mediate some but not all of the association between maternal hyperglycaemia and offspring adiposity. However, the mechanisms underlying this effect are likely to be complex, since TAGs and larger lipid species are unlikely to cross the placenta intact.

#### Neonatal hypoglycaemia and hyperinsulinism at birth (cord C-peptide)

Neonatal hypoglycaemia and cord C-peptide were both associated with metabolite changes from the first trimester, during the period of fetal pancreatic development. Early pregnancy TAG abundance (maternal lipolysis, maternal diet) was strongly associated with neonatal hypoglycaemia, while both lipids and phenols showed associations with cord C-peptide. Previous work suggested that lipid storage is likely to be prioritised over lipid mobilisation in early pregnancy, with lipid mobilisation more common in late pregnancy [19]. The increased abundance of lipid in early pregnancy may be a result of insufficient dietary energy intake (hyperemesis gravidarum), insufficient insulin dosing (lipolysis) or in response to specific dietary patterns (low-carbohydrate diet promoting lipogenesis; direct influence of a high-fat diet). Saccharin and structurally related phenols were consistently related to cord C-peptide. Taken together, these findings suggest that maternal diet in early pregnancy is a key determinant of offspring metabolic health at birth. They also raise the intriguing possibility that offspring beta cell function can be modulated by maternal diet throughout pregnancy.

Although LGA is believed to be related to increased fetal insulin production resulting in increased growth, it had a distinct metabolite profile when compared with neonatal hypoglycaemia and cord blood C-peptide, considered an indirect and direct assessment of neonatal hyperinsulinism, respectively. First, neonatal hypoglycaemia and cord C-peptide both showed changes from the first trimester, while LGA did not. Second, neonatal hypoglycaemia and cord C-peptide both showed prominent increases in TAGs in maternal serum (12 weeks in neonatal hypoglycaemia, 34 weeks in cord C-peptide) which was a less prominent feature of LGA. However, cord blood associations in all three analyses featured carnitines, most prominently in cord blood C-peptide analysis. These differences may suggest that these conditions have true pathophysiological differences, but may also reflect the challenges of suitably measuring these complex conditions, with different time periods of interest. In the analysis of cord blood C-peptide, the outcome of interest is being studied at exactly the same time as the metabolites (also in cord blood). However, LGA develops over months, and neonatal hypoglycaemia may not be evident until 4–24 h after birth.

#### Pre-eclampsia

Pre-eclampsia was associated with increased abundance of PEs, likely from vascular cell membranes in trimesters 1 and 2, suggesting that endothelial damage may be evident from early pregnancy (ESM Appendix 4). The pattern of multiple metabolic changes in pre-eclampsia suggests high energy requirements throughout pregnancy, with utilisation of lipid energy sources in the first trimester, and protein in the second and third trimesters (ESM Appendix 4). Catabolism of protein provides branched-chain amino acids and aromatic amino acids which act as substrates for gluconeogenesis. The involvement of branched-chain amino acids is marked. It is unclear why these would be a preferred substrate for gluconeogenesis, but they have been associated with insulin resistance in other settings [27, 28]. It is also possible that placental insufficiency in pre-eclampsia induces insulin resistance to improve glucose supply to the fetus [29]. Although we had relatively few women with pre-eclampsia in our study, previous work in women without diabetes demonstrated some similarities [30]. However, early pregnancy PCs or phosphatidylserines were most predictive of pre-eclampsia in women without diabetes [31, 32], while PEs were more prominent in our study. Biomarkers derived from general maternity populations may not be equally predictive of suboptimal outcomes in pregnant women with type 1 diabetes.

### Relevance to clinical care and other health policy

#### Relevance to clinical management – insulin dosing and maternal diet

This study demonstrates for the first time a possible metabolic link between maternal hyperglycaemia and offspring adiposity in diabetes pregnancy mediated partially through altered maternal and offspring lipid metabolism. Increases in the abundance of many classes of lipids were common in all analyses performed, likely to be caused by insufficient insulin availability, the key pathophysiological feature of type 1 diabetes. Our work suggests that despite improved access to CGM and insulin pumps, current efforts to address glycaemia do not fully correct the underlying metabolic abnormality.

While it is well established that maternal diet influences insulin dose requirements, our work also identified an association between cord C-peptide and number of metabolite changes in the first trimester (phenols, lipids) which may be associated with maternal dietary intake of sugar substitutes, high-glycaemic-index carbohydrates and phenol compounds. These findings suggest that maternal diet is a key modifiable determinant of offspring health, independently of maternal glycaemia. Future work on optimising insulin dosing from the first trimester, with better matching of insulin to dietary intake and insulin resistance, is needed to improve maternal and neonatal health.

#### Relevance to clinical management – timing of access to care

Traditional approaches to identify and treat women at particular risk of LGA, neonatal hypoglycaemia and pre-eclampsia have focused on mid-to-late pregnancy. Our study suggests that early pregnancy is also a key period in determining outcomes. New strategies are needed to optimise access to care in the first trimester.

### Conclusions

Maternal diet, lipid metabolism, insulin dosing and glycaemia are important modifiable factors in the pathophysiology of perinatal complications in type 1 diabetes pregnancy.

### Supplementary Information

Below is the link to the electronic supplementary material.

Supplementary file1 (PDF 954 KB)

Supplementary file2 (XLSX 23148 KB)

## Data Availability

The data that support the findings of this study are available on request from the CONCEPTT trial steering committee via senior author HRM (Helen.Murphy@uea.ac.uk). The data are not publicly available as they contain information that could compromise research participant privacy/consent.

## Abbreviations

1,5-AG: 1,5-Anhydroglucitol
CGM: Continuous glucose monitoring
Coeff: Standardised coefficient
CONCEPTT: Continuous Glucose Monitoring in Women with Type 1 Diabetes in Pregnancy Trial
DAG: Diacylglycerol
GROW: Gestation Related Optimal Weight
LGA: Large for gestational age
PC: Phosphatidylcholine
PE: Phosphatidylethanolamine
PI: Phosphatidylinositol
TAR: Time-above-range
TAG: Triacylglycerol
TIR: Time-in-range

## Authors’ relationships and activities

HRM is a member of the editorial board of Diabetologia. HRM has received honoraria for speaking engagements from Medtronic, Roche, Novo Nordisk, Eli-Lilly and is a member of the Medtronic European Advisory Board. CLM has received research support from Dexcom Inc. DSF is a member of the Novo Nordisk Expert Panel for the EXPECT trial and has received honoraria for speaking engagements from Medtronic. All other authors declare that there are no relationships or activities that might bias, or be perceived to bias, their contribution to this manuscript.
