# Supplementary material for: Metabolomic insights into maternal and neonatal complications in pregnancies affected by type 1 diabetes
Source: Diabetologia. 2023 Aug 24;66(11):2101–16. doi: 10.1007/s00125-023-05989-2 (PMC10542716; doi:10.1007/s00125-023-05989-2)
Supplement: Supplementary file 1 — Supplementary file1 (PDF 954 KB) [file 125_2023_5989_MOESM1_ESM.pdf]

Supplementary Material

APPENDIX 1 - CONCEPTT Collaborative Group

(Listed according to recruitment numbers):

Cambridge University Hospitals NHS Foundation Trust, Cambridge, UK: Helen Murphy, Jeannie Grisoni, Carolyn Byrne, Sandra Neoh, Katy Davenport, (43); Alberta Health Services, University of Calgary, Calgary, Canada: Lois Donovan, Claire Gougeon, Carolyn Oldford, Catherine Young (39); King's College Hospital, London, UK: Stephanie Amiel, Katharine Hunt, Louisa Green, Helen Rogers, Benedetta Rossi (29); Mount Sinai Hospital, Toronto, Canada: Denice Feig, Barbara Cleave, Michelle Strom (22); Hospital de la Santa Creu i Sant Pau, Barcelona, Spain and CIBER-BBN, Zaragoza, Spain: Rosa Corcoy, Alberto de Leiva, Juan María Adelantado, Ana Isabel Chico, Diana Tundidor (22); The Ottawa Hospital General Campus, Ottawa, Canada: Erin Keely, Janine Malcolm, Kathy Henry (15); Ipswich Hospital NHS Trust, Ipswich, UK: Damian Morris, Gerry Rayman, Duncan Fowler, Susan Mitchell, Josephine Rosier (13); Norfolk and Norwich University Hospital, Norwich, UK: Rosemary Temple, Jeremy Turner, Gioia Canciani, Niranjala Hewapathirana, Leanne Piper (13); St. Joseph's Health Centre, London, Canada: Ruth McManus, Anne Kudirka, Margaret Watson (13); Niguarda ca' Granda Hospital, Milano, Italy: Matteo Bonomo, Basilio Pintaudi, Federico Bertuzzi, Giuseppina Daniela Corica, Elena Mion (12); Sunnybrook Health Sciences Centre, Toronto, Canada: Julia Lowe, Ilana Halperin, Anna Rogowsky, Sapida Adib (11); Glasgow Royal Infirmary, Glasgow, UK: Robert Lindsay, David Carty, Isobel Crawford, Fiona Mackenzie, Therese McSorley (10); McMaster University, Hamilton, Canada: John Booth, Natalia McInnes, Ada Smith, Irene Stanton, Tracy Tazzeo (8); Centre hospitalier universitaire de Québec, Quebec City, Canada: John Weisnagel (6); Queen's Medical Centre, Nottingham, UK: Peter Mansell, Nia Jones, Gayna Babington, Dawn Spick (6); Royal Victoria Infirmary, Newcastle Upon Tyne, Newcastle, UK: Malcolm MacDougall, Sharon Chilton, Terri Cutts, Michelle Perkins (6); Leeds Teaching Hospitals NHS Trust, Leeds, UK: Eleanor Scott, Del Endersby (6); Royal Infirmary of Edinburgh, Edinburgh, UK: Anna Dover, Frances Dougherty, Susan Johnston (6); Sheffield Teaching Hospitals NHS Foundation Trust, Sheffield, UK: Simon Heller, Peter Novodorsky, Sue Hudson, Chloe Nisbet (6); Izaak Walton Killam Health Sciences Centre (IWK), Halifax, Canada: Thomas Ransom, Jill Coolen, Darlene Baxendale (5); University Hospital Southampton NHS Foundation Trust, Southampton, UK: Richard Holt, Jane Forbes, Nicki Martin, Fiona Walbridge (6); Galway University Hospitals, Galway, Ireland: Fidelma Dunne, Sharon Conway, Aoife Egan, Collette Kirwin (4); Central Manchester University Hospitals NHS Foundation Trust, Manchester, UK: Michael Maresh, Gretta Kearney, Juliet Morris, Susan Quinn (4); South Tees Hospitals, NHS Foundation Trust, Middlesbrough, UK: Rudy Bilous, Rasha Mukhtar (4); Centre de Recherche du Centre Hospitalier de Université de Montréal (CR-CHUM), Montreal, Canada: Ariane Godbout, Sylvie Daigle (3); The Dudley Group NHS FT, Russells Hall Hospital, Dudley, UK: Alexandra Lubina Solomon, Margaret Jackson, Emma Paul, Julie Taylor (3); Kingston General Hospital, Queen's University, Kingston, Canada: Robyn Houlden, Adriana Breen (3); Guys and St Thomas' NHS Foundation Trust, London, UK: Anita Banerjee, Anna Brackenridge, Annette Briley, Anna Reid, Claire Singh (2); Royal University Hospital, Saskatoon, Canada: Jill Newstead-Angel, Janet Baxter (2); Grampian Diabetes Centre, Aberdeen, UK: Sam Philip, Martyna Chlost, Lynne Murray (2); William Sansum Diabetes Center, Santa Barbara, USA: Kristin Castorino, Lois Jovanovic, Donna Frase (2). The Centre for Clinical Trial Support (CCTS) at the Sunnybrook Research Institute, Toronto, Canada: Sonya Mergler, Kathryn Mangoff, Johanna Sanchez, and Gail Klein. The Jaeb Center for Health Research, Tampa, USA: Katrina Ruedy and Craig Kollman. Juvenile Diabetes Research Foundation (non-clinical collaborators): Olivia Lou and Marlon Pragnell.

APPENDIX 2: MATERNAL BMI, GESTATIONAL WEIGHT GAIN AND MATERNAL DIET

Metabolomic and lipidomic changes associated with maternal BMI

Baseline characteristics and pregnancy outcomes of women categorised according to enrolment BMI are shown in ESM Table 1. There were no significant differences in LGA rates between lean (38/90; 42.2%) and overweight/obese (30/84; 35.7%) women.

Maternal BMI at study enrolment was significantly associated with multiple metabolites in maternal serum, especially at 12 and 24 weeks (ESM Figure A2.1; ESM Table 1-2). At 12 weeks, there were significant positive associations with amino acid derivatives and negative associations with progesterone metabolites, 3beta-hydroxy-5-cholestenoate (bile acid synthesis), carotene diol, phenylpropionate and several PCs. At 24 weeks, negative associations with progesterones and carotene diol remained; effects upon phospholipids were more marked. There were negative associations with hexosylceramide (HCER(24:0)), seven phosphatidylcholine species (mostly containing polyunsaturated fatty acids FA(18:2) or FA(22:6) and six PEs, including plasmalogens (many containing FA(18:2); linoleic acid, n-6). There were positive associations with another ceramide (DCER(20:1)) and with Fibrinopeptides A (3-15) and A (8-16) (exact species identification unconfirmed). At 34 weeks, there were no positive associations between maternal metabolites and maternal BMI, but there were negative associations with carotene diol and PC(18:2/20:1).

Maternal BMI was associated with multiple changes in the cord blood metabolome. There were positive associations with cholesteryl esters, some phospholipids (PC, PI, SMs) and ten triglycerides (containing 52-54 carbons and 2-5 double bonds; all likely to contain FA(18:2)). There were negative associations with a smaller cholesteryl ester (CE(16:1)), a lyso-phosphatidylethanolamine (LPE(20:2)), and numerous phospholipids (PCs, PEs and PE plasmalogens, PIs) and several triglycerides. Almost all lipid species in cord blood with negative associations to maternal BMI contained fatty acids FA(16:0), FA(18:0) or FA(18:1).

Metabolomic and lipidomic changes associated with gestational weight gain (ESM Figure A2.2, ESM Table 3 and 4)

Data on gestational weight gain from enrolment to 34 weeks was available on 141 women. Baseline characteristics and pregnancy outcomes of women categorised according to the presence or absence of excessive gestational

weight gain (using Institute of Medicine thresholds) are shown in ESM Table 3. As expected, the presence of excessive gestational weight gain influenced the maternal metabolome only in late pregnancy. At 34 weeks there was a positive association with PC(18:0/18:3) and a negative association with alpha-tocopherol. The cord blood metabolome showed no associations with maternal excessive gestational weight gain.

Metabolomic and lipidomic changes associated with habitual diet (ESM Figure A2.3, ESM Table 5-8)

Baseline characteristics and pregnancy outcomes of women categorised according to dietary practices are shown in ESM Tables 5 and 7. Dietary information was available on 56 women at 12 weeks. Participants reported consuming mean 1652 kcal/day (SD 451.5; median 1729 kcal/day) with a macronutrient composition of 43.9% (7.3) energy from carbohydrates, 37.4% (6.4) from fat and 17.5% (4.2) from protein. Maternal total energy intake was positively associated with piperine sulfate (found in black pepper) at 12 weeks (ESM Figure A2.3). Maternal carbohydrate intake (% of total energy intake) was positively associated with CE(16:1). Maternal protein intake (% of total energy intake) was positively associated with two PE plasmalogens, and negatively associated with monoglyceride (18:1). Maternal fat intake (% of total energy intake) showed negative associations with a cholesteryl ester, a lyso-phosphatidylcholine (LPC(16:1)), a lyso-phosphatidylethanolamine (LPE(16:1)), and multiple PCs, PIs and triglycerides containing fatty acids (16:0) and (16:1). Women consuming a higher energy diet (energy intake above the median) had a slightly lower HbA1c at 12 weeks but no significant differences in pregnancy outcomes compared to women consuming a lower energy diet (ESM Table 5). A comparison of women who consumed a low-carbohydrate high-fat diet (LCHF; average 35% carbohydrate, 45% fat and 20% protein) demonstrated no differences in glycaemia or outcomes, except for increased neonatal hypoglycaemia on adjusted regression analysis ESM Table 7). Small numbers in the dietary analysis preclude definitive conclusions.

Maternal BMI was not significantly associated with maternal intake of fat, other macronutrients or polyunsaturated fatty acids (ESM Table 1). Maternal intake of retinol or carotene was not associated with maternal BMI on unadjusted analysis or when adjusted for total maternal energy intake.

ESM Figure A2.1 – Metabolites in maternal blood at 12, 24 and 34 weeks' gestation and in cord blood at birth in association with maternal BMI in early pregnancy (measured at first visit, approx. 8 -9 weeks' gestation).

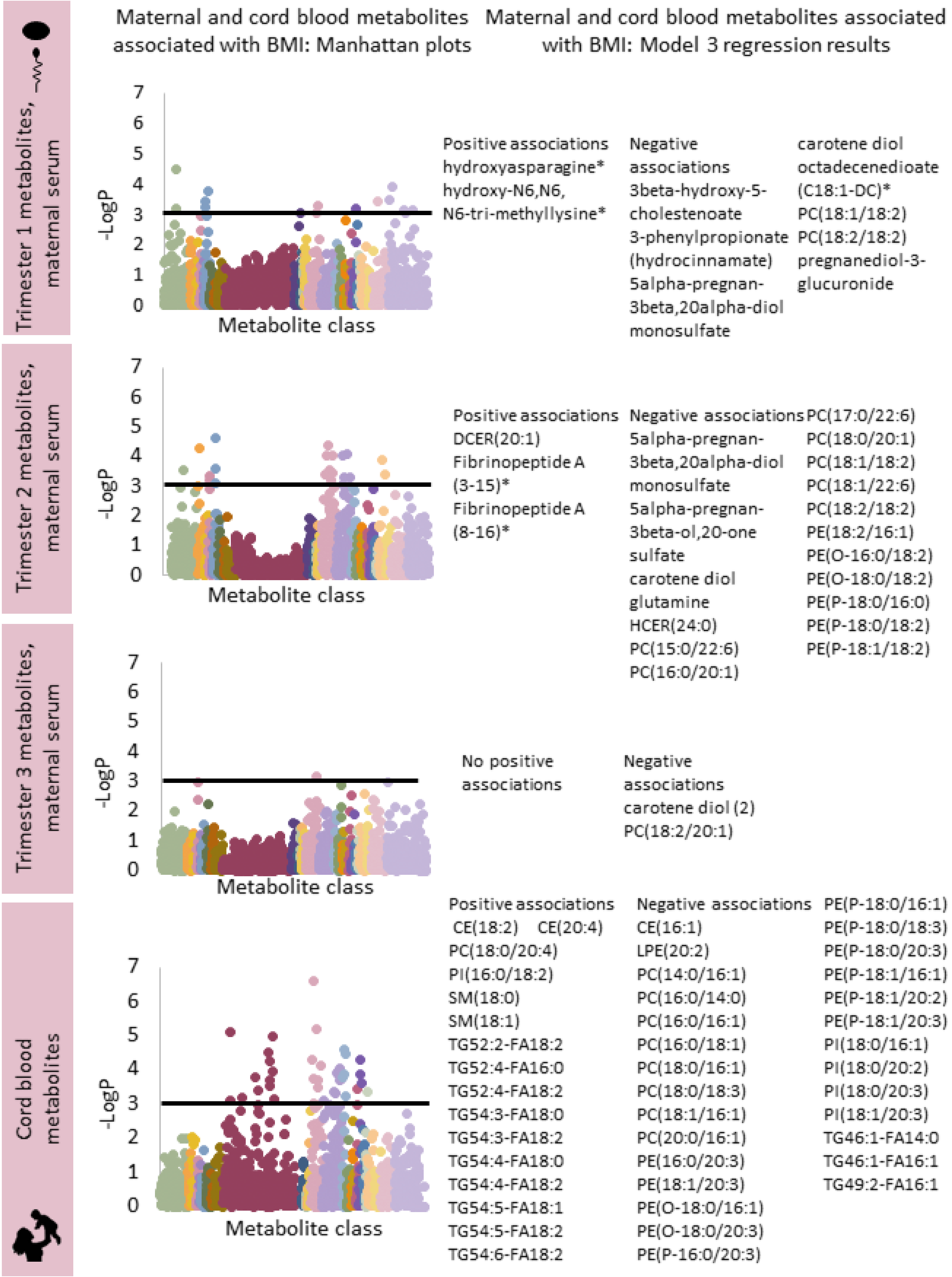

ESM Figure A2.2 – Metabolites in maternal blood at 12, 24 and 34 weeks' gestation and in cord blood at birth in association with gestational weight gain.

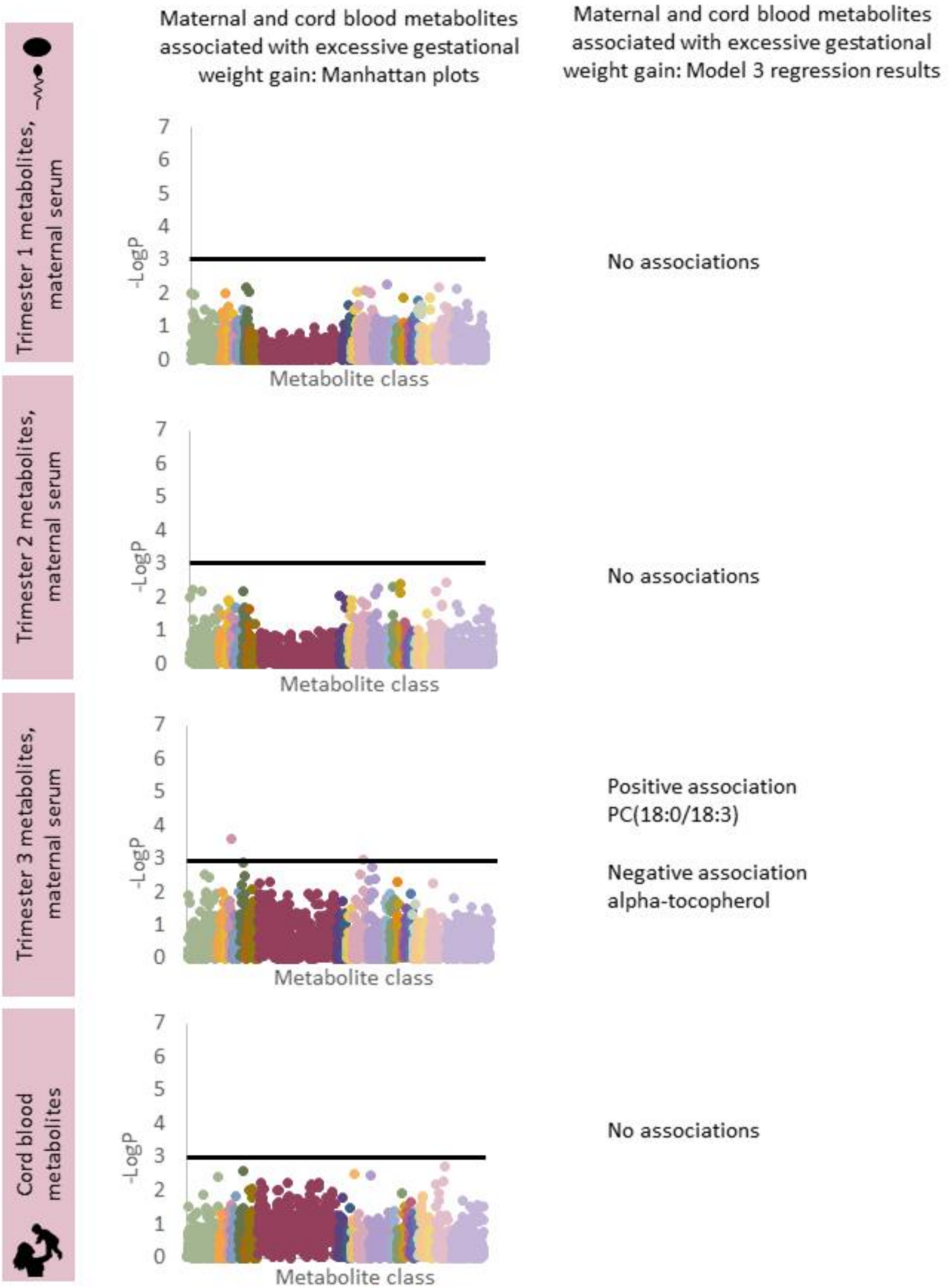

ESM Figure A2.3 – Metabolites in maternal blood at 12 weeks' gestation in association with maternal self-reported diet at 12 weeks' gestation.

A: Maternal total energy intake (kilocalories) at 12 weeks and metabolites in maternal serum at 12 weeks, adjusted (M3)

Trimester 1 metabolites, maternal serum

Positive associations
sulfate of piperine metabolite C18H21NO3 (1)*

No negative associations

B: Maternal carbohydrate intake (% of total calories) at 12 weeks and metabolites in maternal serum at 12 weeks, adjusted (M3)

Trimester 1 metabolites, maternal serum

Positive associations
CE(16:1)

No negative associations

C: Maternal protein intake (% of total calories) at 12 weeks and metabolites in maternal serum at 12 weeks, adjusted (M3)

Trimester 1 metabolites, maternal serum

Positive associations
PE(P-16:0/18:2)
PE(P-18:0/18:2)

Negative associations
MAG(18:1)

D: Maternal fat intake (% of total calories) at 12 weeks and metabolites in maternal serum at 12 weeks, adjusted (M3)

Trimester 1 metabolites, maternal serum

No positive associations

Negative associations
CE(18:4)
LPC(16:1)
LPE(16:1)
PC(15:0/16:1)
PC(16:0/12:0)
PC(16:0/14:0)
PC(16:0/16:1)
PC(16:0/18:1)
PC(17:0/20:5)
PI(16:0/16:0)
PI(16:0/20:3)
PI(18:0/16:1)
PI(18:1/16:1)
TG48:1-FA16:1
TG48:2-FA16:1

Maternal analyses adjusted for maternal TIR, age, ethnicity, parity, education and intervention.
N=20-44. Cord blood analyses not included – insufficient numbers. Limit of significance $p \leq 0.0011$.

APPENDIX 3: METABOLITE CHANGES ASSOCIATED WITH OFFSPRING ADIPOSITY, INCLUDING MEDIATION ANALYSIS

ESM Figure A3.1 – Metabolites in maternal blood at 12, 24 and 34 weeks' gestation and in cord blood at birth in association with adiposity (sum of four skinfolds) in offspring.

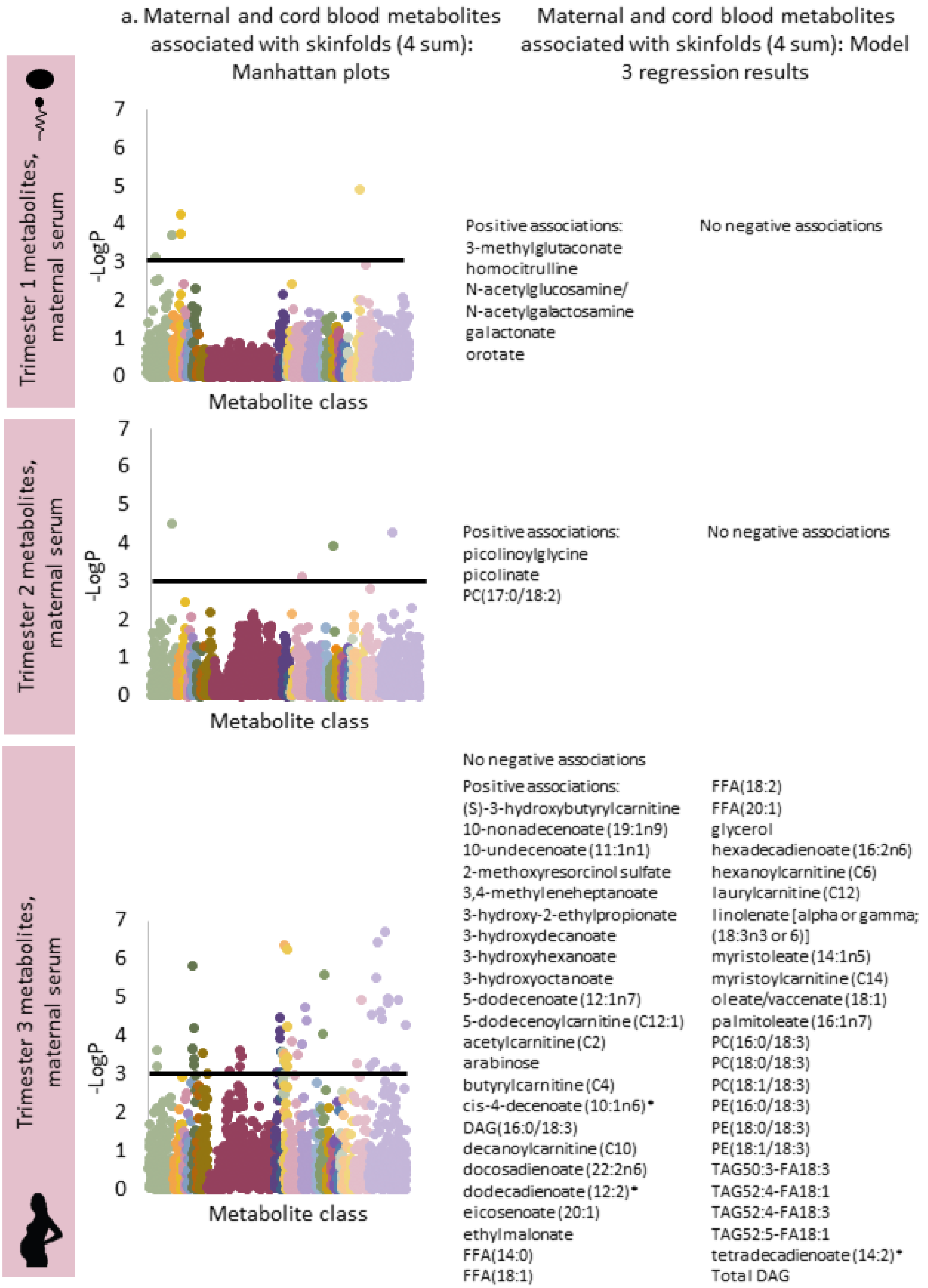

ESM Figure A3.1 – continued

b. Maternal and cord blood metabolites associated with skinfolds (4 sum): Manhattan plots

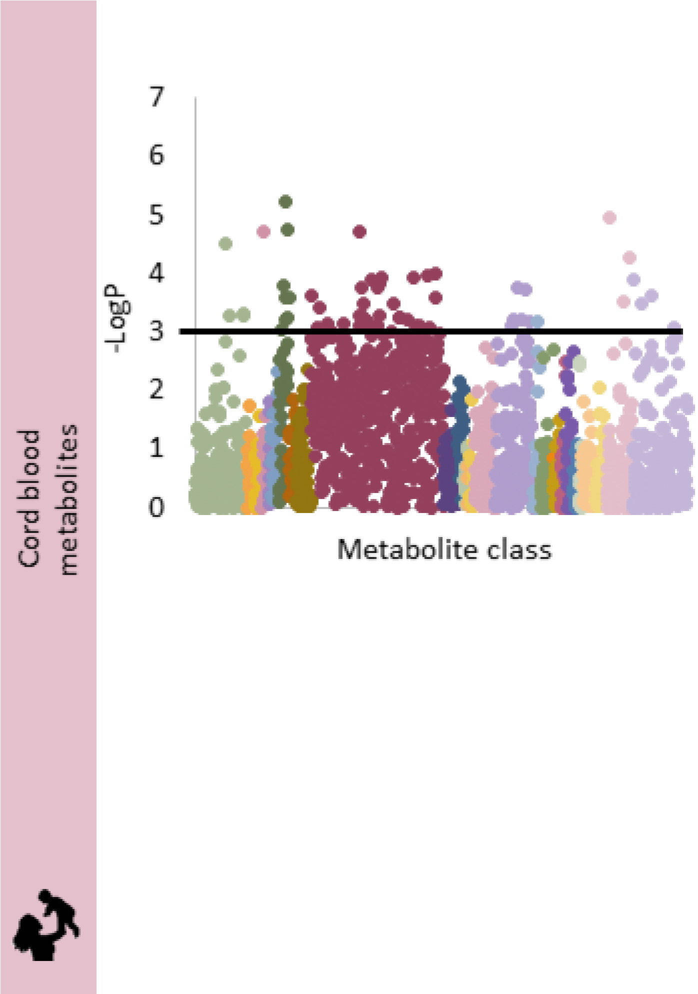

Maternal and cord blood metabolites associated with skinfolds (4 sum): Model 3 regression results

Negative associations: PE(18:2/22:4)

Positive associations:
2-hydroxyhippurate (salicylurate)
adenosine 5'-diphosphoribose (ADP-ribose)
arachidoylcarnitine (C20)*
eicosenoylcarnitine (C20:1)*
iminodiacetate (IDA)
linolenoylcarnitine (C18:3)*
margaroylcarnitine (C17)*
myristoylcarnitine (C14)
N,N,N-trimethyl-5-aminovalerate
N-acetylaspartate (NAA)
oleoylcarnitine (C18:1)
palmitoylcarnitine (C16)
PE(O-16:0/18:1)
PE(O-18:0/18:1)
PE(P-16:0/18:1)
PE(P-18:0/18:1)
PE(P-18:1/18:1)
PI(18:0/20:3)
salicylate
sarcosine
spermidine
stearoylcarnitine (C18)
TAG36:0-FA12:0
TAG44:1-FA14:0
TAG44:2-FA16:0
TAG45:0-FA15:0
TAG46:0-FA12:0
TAG47:0-FA14:0
TAG47:0-FA17:0
TAG47:2-FA18:1
TAG49:1-FA16:1
TAG51:0-FA18:0
TAG51:1-FA15:0
TAG51:1-FA18:0
TAG51:2-FA18:2
TAG51:3-FA15:0
TAG51:3-FA17:0
TAG51:4-FA18:3
TAG52:1-FA20:1
TAG52:3-FA14:0
TAG52:3-FA20:0
TAG52:4-FA22:1
TAG52:4-FA22:4
TAG52:5-FA20:3
TAG52:5-FA20:5
TAG52:7-FA16:0
TAG54:0-FA18:0
TAG54:2-FA20:0
TAG54:4-FA18:3
TAG54:4-FA22:1
TAG54:7-FA18:3
TAG55:7-FA22:6
TAG56:2-FA16:0
TAG56:4-FA18:2
TAG56:7-FA20:3
TAG56:8-FA16:1
TAG58:10-FA22:5
TAG58:6-FA16:0
TAG58:7-FA22:4

ESM Figure A3.2 – Adiposity mediation analysis: do triglycerides mediate the relationship between maternal hyperglycaemia and offspring adiposity?

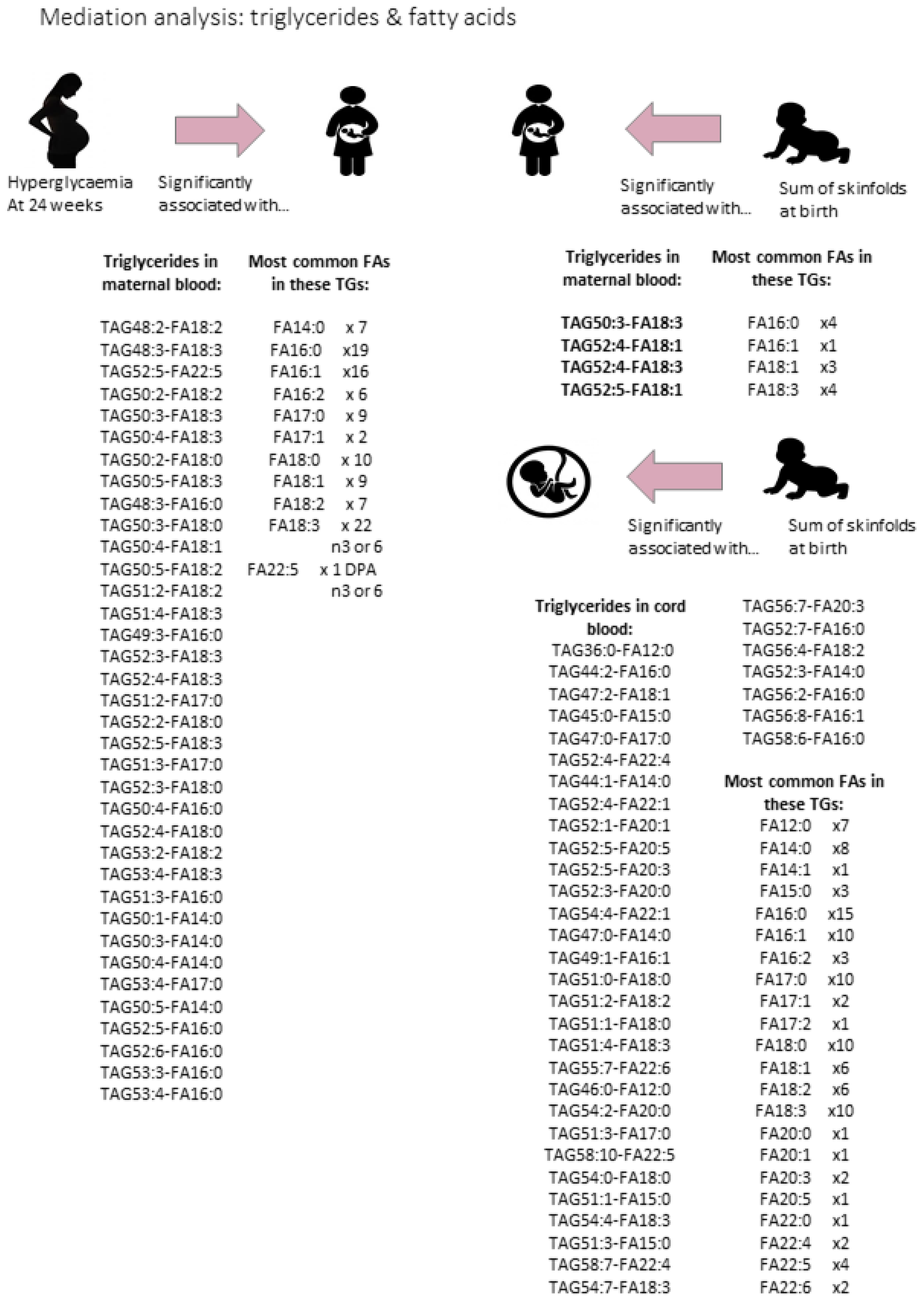

Question:

Do triglycerides in maternal blood mediate the relationship between maternal hyperglycaemia and offspring adiposity?

Regression of effect of maternal hyperglycaemia (CGM time above range) at 24 weeks upon skinfold sum:

Coeff 16.20 (10.57 to 21.83); p=9.28 x 10^{-8}.

Regression of effect of maternal hyperglycaemia at 24 weeks upon skinfold sum, including all listed maternal triglycerides in ESM Figure A2.2:

Coeff 15.24 (7.72 to 22.76); p=0.000131

Regression of effect of maternal glycaemia at 34 weeks upon skinfold sum:

Regression of effect of maternal hyperglycaemia at 34 weeks upon skinfold sum

Coeff 17.20 (9.66 to 24.75); p=0.0000166 (1.66 x 10^{-5}).

Regression of effect of maternal hyperglycaemia at 24 weeks upon skinfold sum, including all listed maternal triglycerides in ESM Figure A2.2:

Coeff 13.74 (2.66 to 24.84); p=0.0160

Question:

Do triglycerides in CORD blood mediate the relationship between maternal hyperglycaemia and offspring adiposity?

Regression of effect of maternal glycaemia at 24 weeks upon skinfold sum:

Regression of effect of maternal hyperglycaemia at 24 weeks upon skinfold sum

Coeff 16.20 (10.57 to 21.83); p=9.28 x 10^{-8}.

Regression of effect of maternal hyperglycaemia at 24 weeks upon skinfold sum, including all listed cord blood triglycerides

Coeff 22.16 (9.50 to 34.81); p=0.00132

Regression of effect of maternal glycaemia at 34 weeks upon skinfold sum:

Regression of effect of maternal hyperglycaemia at 34 weeks upon skinfold sum:

Coeff 17.20 (9.66 to 24.75); p=0.0000166 (1.66 x 10^{-5}).

Regression of effect of maternal hyperglycaemia at 24 weeks upon skinfold sum, including all listed cord blood triglycerides

Coeff 18.27 (0.91 to 35.64); p=0.0401

Interpretation:

Adjustment for specific maternal or cord blood triglycerides markedly reduces the significance of the association between maternal hyperglycaemia and offspring adiposity at 24 weeks and attenuates associations at 34 weeks. This

suggests that triglyceride and related species have a key role in mediating the relationship between maternal hyperglycaemia and offspring body composition. However, adjusting for triglycerides did not consistently attenuate the association, suggesting that glucose itself and/or other mediators may also play a role.

A note on methods and limitations: studying the physiology and pathophysiology of human pregnancy is challenging. We present our mediation analysis in order to extend the body of knowledge and yield new testable hypotheses. However, lipids are highly inter-related, which causes some issues for a mediation analysis. For example, fatty acids can transfer easily from triglycerides to diglycerides and monoglycerides, or exist as free fatty acids in the blood. Free fatty acids can quickly be incorporated into phospholipids and other larger lipid structures. This means that any findings related to triglycerides need to be interpreted as part of a broad overview of lipid homoeostasis at any one time.

Furthermore, the physiology of lipid transfer across the placenta remains imperfectly understood. While it is tempting to assume that triglycerides passing through the placenta are directly mediating these effects, it is likely that fatty acids, but not assembled triglycerides, can pass through the placenta. Fatty acids may reassemble into triglycerides on the fetal side of the placenta. However, fetal triglycerides are also likely to arise from fetal de novo lipogenesis, making new fat, which is likely to be crucial part of early development.

ESM Figure A3.3 – Adiposity mediation analysis: do plasmalogens and carnitines mediate the relationship between maternal hyperglycaemia and offspring adiposity?

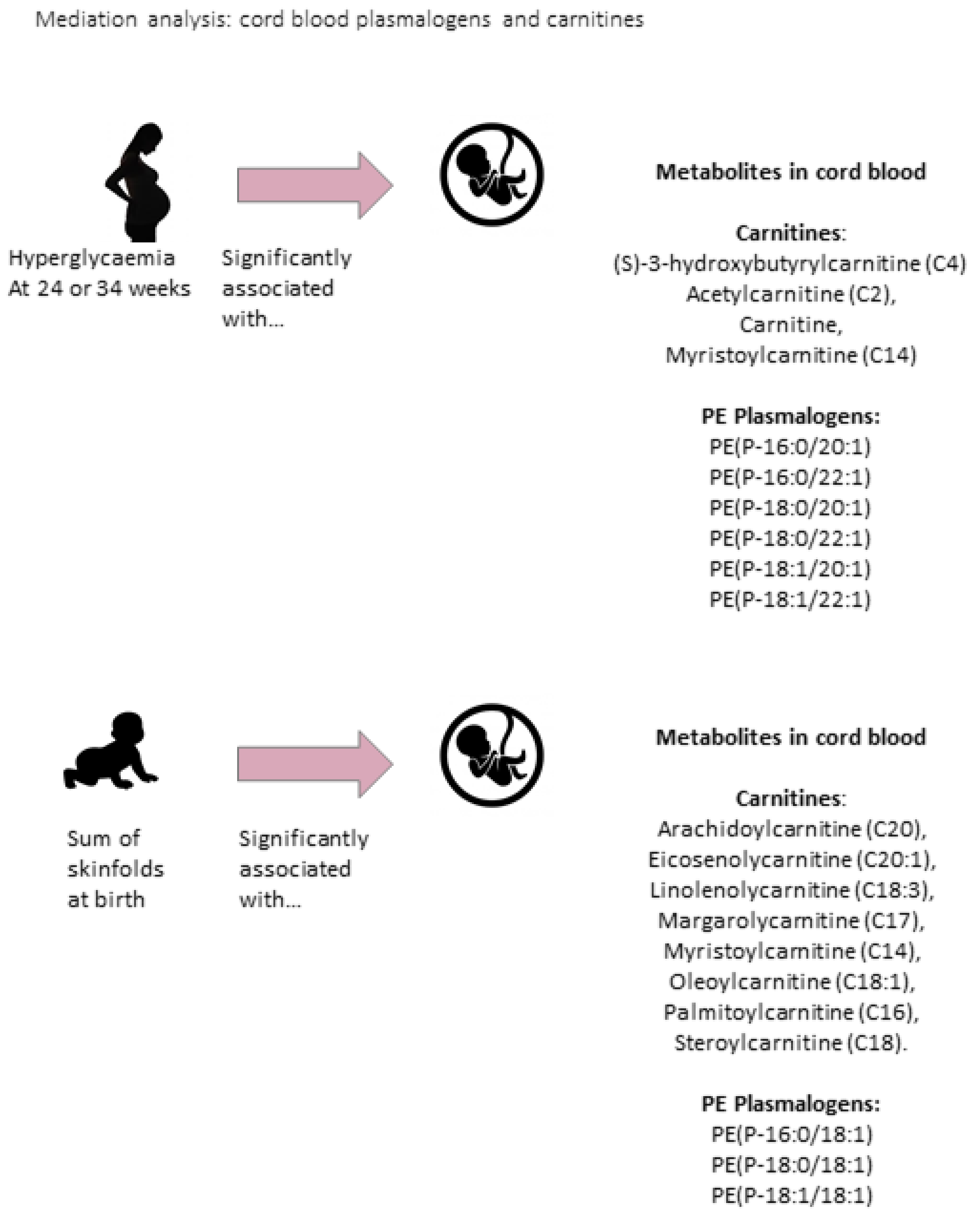

Question:

Do PE plasmalogens mediate the relationship between maternal hyperglycaemia and offspring adiposity?

Regression of effect of maternal glycaemia at 24 weeks upon skinfold sum

Regression of effect of maternal glycaemia at 24 weeks upon skinfold sum:

Coeff 16.20 (10.57 to 21.83); $p=9.28 \times 10^{-8}$.

Regression of effect of maternal glycaemia at 24 weeks upon skinfold sum (all plasmalogens listed above in ESM Figure A3.3 included)

Coeff 12.96 (5.68 to 20.23); p=0.00077.

Regression of effect of maternal glycaemia at 34 weeks upon skinfold sum

Regression of effect of maternal glycaemia at 34 weeks upon skinfold sum:

Coeff 17.20 (9.66 to 24.75); p=0.0000166 (1.66×10^{-5}).

Regression of effect of maternal glycaemia at 34 weeks upon skinfold sum (all plasmalogens listed above in ESM Figure A3.3 included):

Coeff 8.22 (-2.26 to 18.71); p=0.121.

Question:

Do carnitines mediate the relationship between maternal hyperglycaemia and offspring adiposity?

Regression of effect of maternal glycaemia at 24 weeks upon skinfold sum

Regression of effect of maternal glycaemia at 24 weeks upon skinfold sum:

Coeff 16.20 (10.57 to 21.83); p=9.28×10^{-8}.

Regression of effect of maternal glycaemia at 24 weeks upon skinfold sum (all carnitines mentioned above in ESM Figure A3.3 included in the model):

Coeff 13.70 (5.69 to 21.72); p=0.001197

Regression of effect of maternal glycaemia at 34 weeks upon skinfold sum

Regression of effect of maternal glycaemia at 34 weeks upon skinfold sum:

Coeff 17.20 (9.66 to 24.75); p=0.0000166 ($1.66*10^{-5}$).

Regression of effect of maternal glycaemia at 24 weeks upon skinfold sum (all carnitines mentioned above in ESM Figure A3.3 included in the model):

Coeff 10.87 (-1.16 to 22.90); p=0.076

Question:

Could both carnitines and plasmalogens mediate the relationship between maternal hyperglycaemia and offspring adiposity?

Regression of effect of maternal glycaemia at 24 weeks upon skinfold sum

Regression of effect of maternal glycaemia at 24 weeks upon skinfold sum:

Coeff 16.20 (10.57 to 21.83); p=9.28×10^{-8}.

Regression of effect of maternal glycaemia at 24 weeks upon skinfold sum (all carnitines and plasmalogens mentioned above in ESM Figure A3.3 included in the model):

Coeff 12.47 (3.26 to 21.67); p=0.00917

Regression of effect of maternal glycaemia at 34 weeks upon skinfold sum

Regression of effect of maternal glycaemia at 34 weeks upon skinfold sum:

Coeff 17.20 (9.66 to 24.75); p=0.0000166 (1.66×10^{-5}).

Regression of effect of maternal glycaemia at 24 weeks upon skinfold sum (all carnitines and plasmalogens mentioned above in ESM Figure A3.3 included in the model):

Coeff 5.97 (-6.89 to 18.83) p=0.353

Interpretation:

Carnitines and PE plasmalogens appear to be important mediators of the relationship between maternal hyperglycaemia and offspring adiposity. However, they do not appear to be the sole mediators of this relationship as they do not consistently obliterate the significant relationship between hyperglycaemia and skinfold sum.

APPENDIX 4: METABOLITE CHANGES ASSOCIATED WITH PREECLAMPSIA

ESM Figure A4.1: Availability of samples for maternal blood (a) and cord blood (b) analysis.

LGA: large for gestational age.

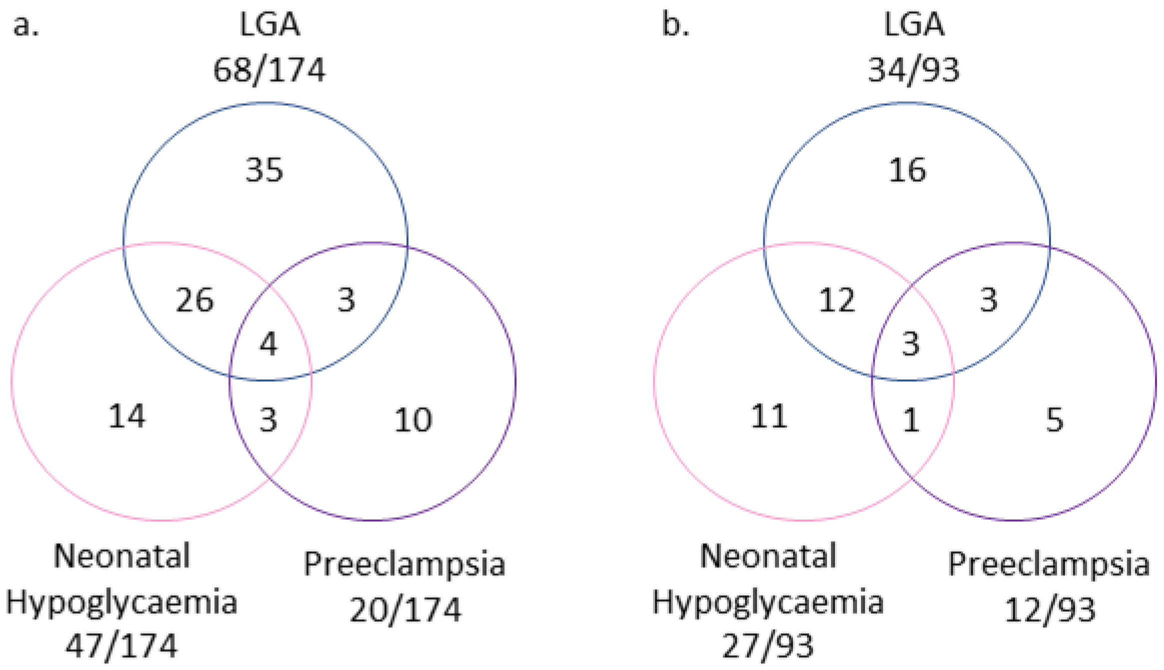

ESM Figure A4.2: Maternal and cord blood metabolites associated with preeclampsia (See manuscript text for description).

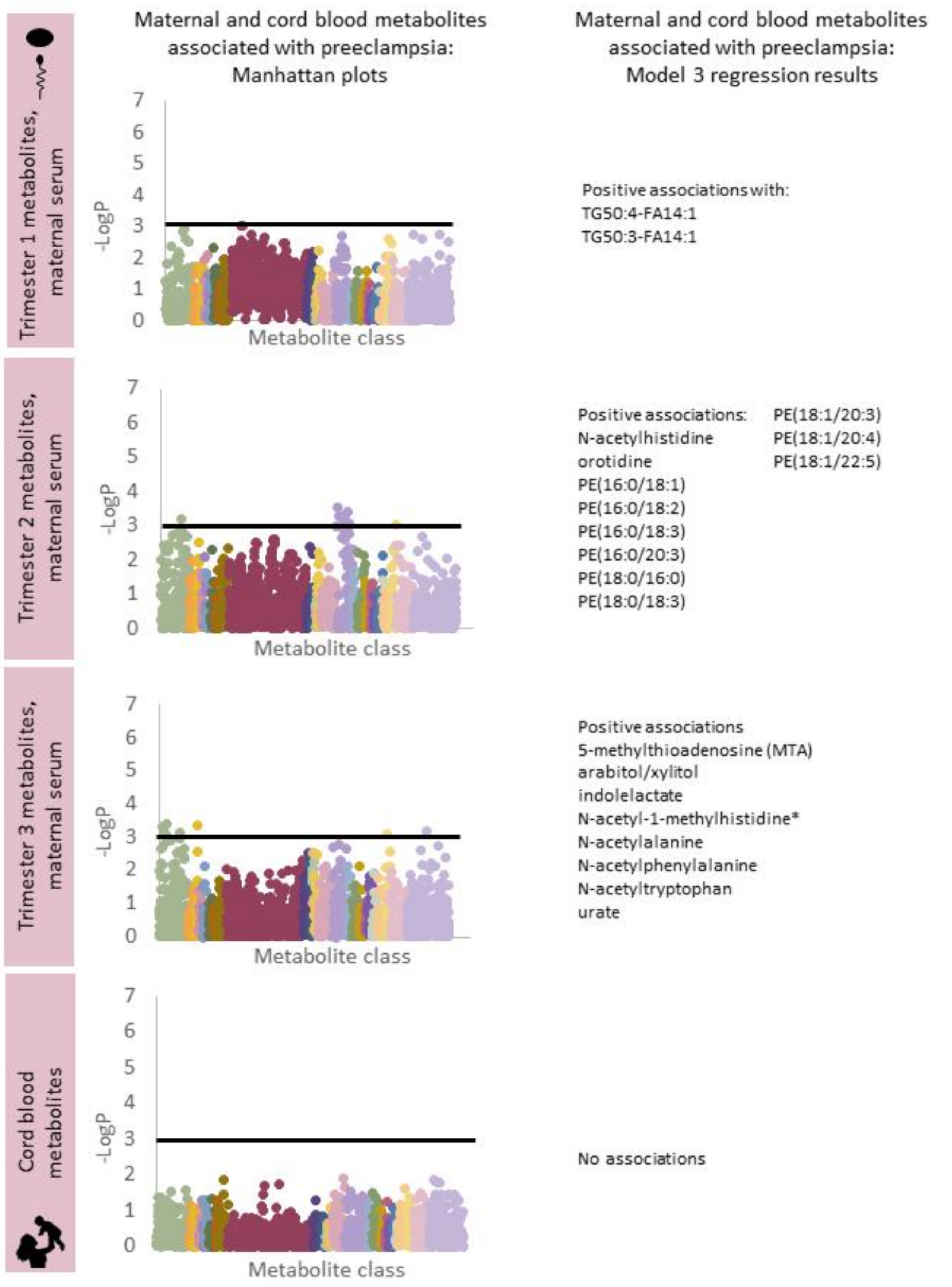

Supplementary Tables

Please see separate file for supplementary tables.
